# A case of surgical treatment for bronchial foreign bodies with obstructive pneumonia

**DOI:** 10.1002/rcr2.1325

**Published:** 2024-03-19

**Authors:** Yuki Yamashita, Motoi Ugajin, Saki Yanoma, Masakatsu Yamashita, Hisanori Kani

**Affiliations:** ^1^ Department of Respiratory Medicine Nagoya Tokushukai General Hospital Kasugai Japan; ^2^ Department of Thoracic Surgery Nagoya Tokushukai General Hospital Kasugai Japan

**Keywords:** bronchial foreign body, case report, obstructive pneumonia, root canal filling, surgical therapy

## Abstract

Children and older adults are prone to unintentional foreign body aspiration. A 69‐year‐old man with fever and anorexia presented with obstructive pneumonia resulting from foreign body aspiration. Attempts to remove the foreign body using a bronchoscope failed due to its adhesion to the periphery of the bronchus. Although antibiotic therapy did not improve the obstructive pneumonia caused by the bronchial foreign body, surgery enabled an improvement. The surgical specimen showed similar pathological findings as the fine brown granular material observed in root granulomas occurring as a complication following leakage of root canal filling used in the treatment of dental caries. Therefore, the bronchial foreign body may have been a dental filling. Case reports describing surgical improvement of difficult‐to‐remove bronchial foreign bodies with concurrent infection are rare.

## Graphical abstract

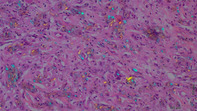

## INTRODUCTION

Children and older adults are at a risk of unintentional foreign body aspiration.^1^, ^2^ If symptoms such as coughing or dyspnoea are present, immediate consultation with a physician is advised. However, older adults may be unaware of aspirating a foreign body due to a diminished cough reflex or drug‐induced dysphagia. Typically, a bronchial foreign body can be removed using a bronchoscope immediately after aspiration. However, if a foreign body remains unnoticed for a long time, it may become lodged in the bronchus, rendering removal challenging. Duan et al. published a review of 23 patients who underwent surgical treatment for late‐diagnosed bronchial foreign body aspiration at their hospital between 1980 and 2010.^3^ Herein, we present a case of a foreign body in the airway of an older adult that could not be removed using bronchoscopy and required surgery.

## CASE REPORT

**FIGURE 1:**
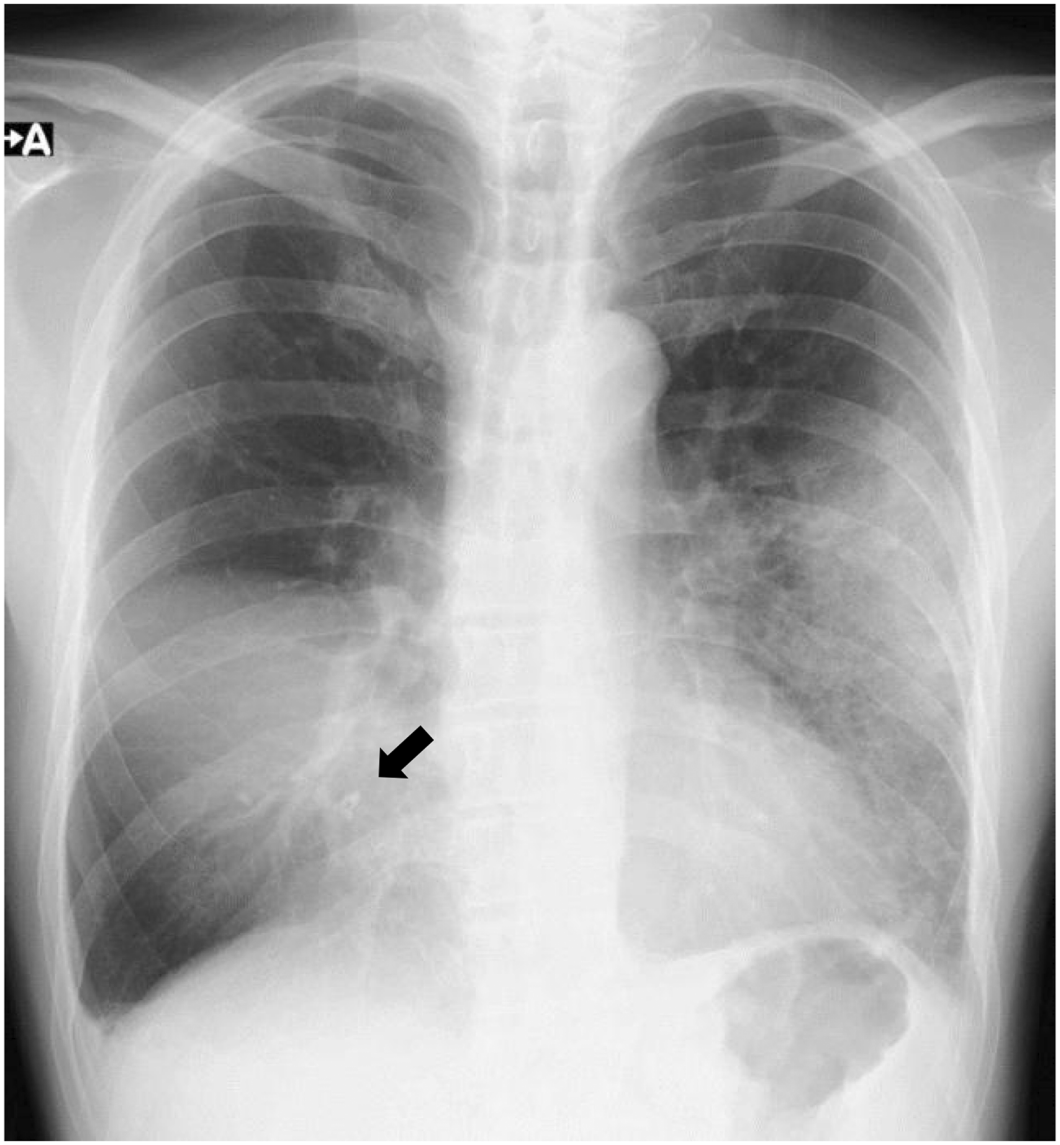
Chest radiograph showing right middle lobe atelectasis, an artefact suggestive of a bronchial foreign body in the right lower lung field (arrow), and an infiltration shadow in the left lower lung field.

**FIGURE 2:**
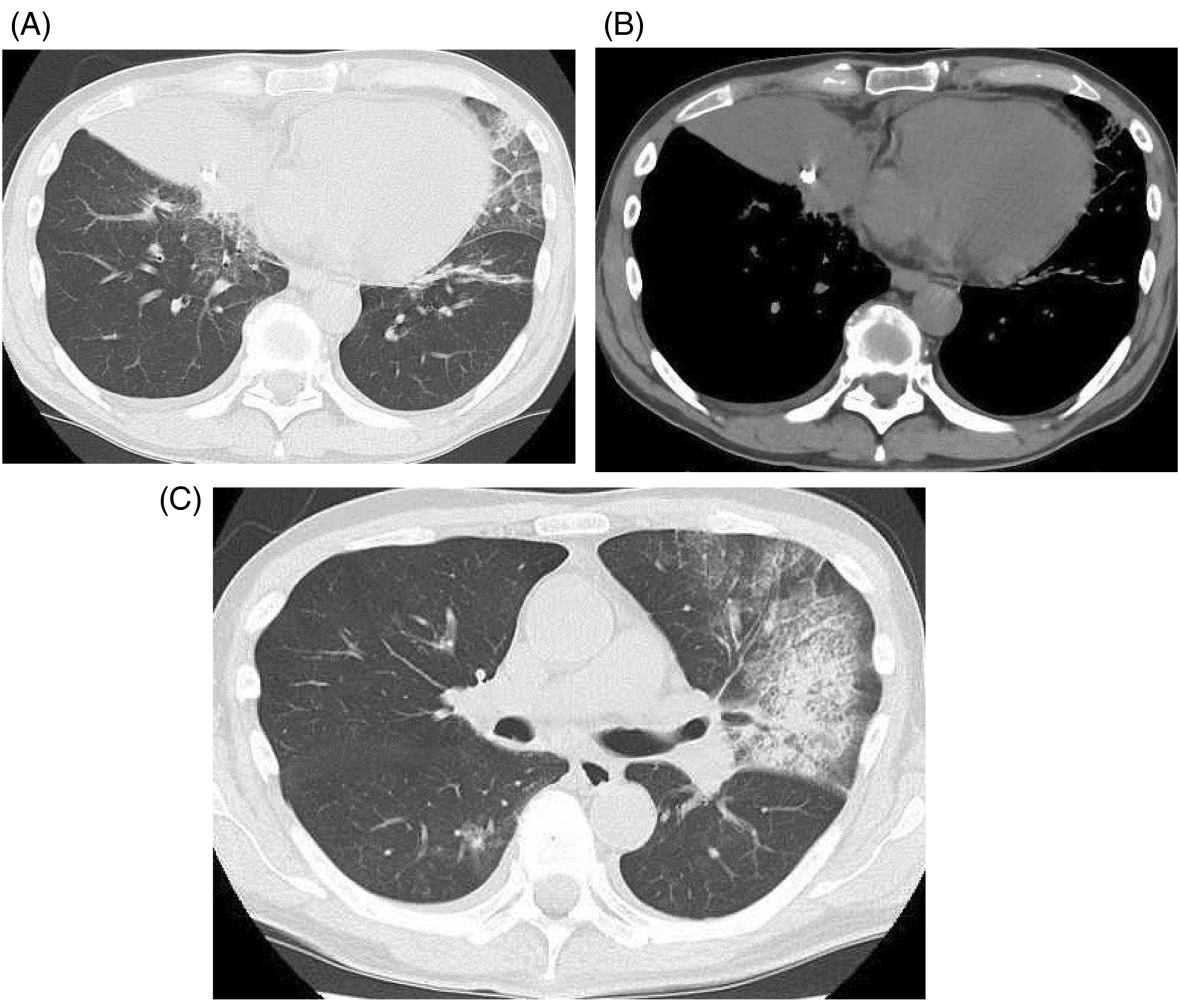
Chest computed tomography at the first visit shows right middle lobe atelectasis, an artefact stuck in the right middle lobe bronchus (A and B), and an infiltration shadow in the lingular segment of the left lung (C).

A 69‐year‐old man presented to our hospital with fever, dyspnoea, drowsiness, and loss of appetite for several weeks. Chest exam revealed decreased breath sounds on the right side. Chest radiography revealed right middle lobe atelectasis, an artefact suggestive of a bronchial foreign body in the right lower lung field, and an infiltration shadow in the left lower lung field (Figure 1). Chest computed tomography (CT) indicated right middle lobe atelectasis, an artefact stuck in the right middle lobe bronchus, and an infiltration shadow in the lingular segment of the left lung (Figure 2A–C). The patient had a history of manic depression and insomnia and was regularly taking sleeping pills and psychotropic drugs (including lithium carbonate, nitrazepam, brotizolam, zopiclone, olanzapine, eszopiclone, quetiapine, and lorazepam), which were assumed to have impaired swallowing, prompting unintentional foreign body aspiration. Chest radiography indicated that the patient had aspirated a fragment of a denture.

**FIGURE 3:**
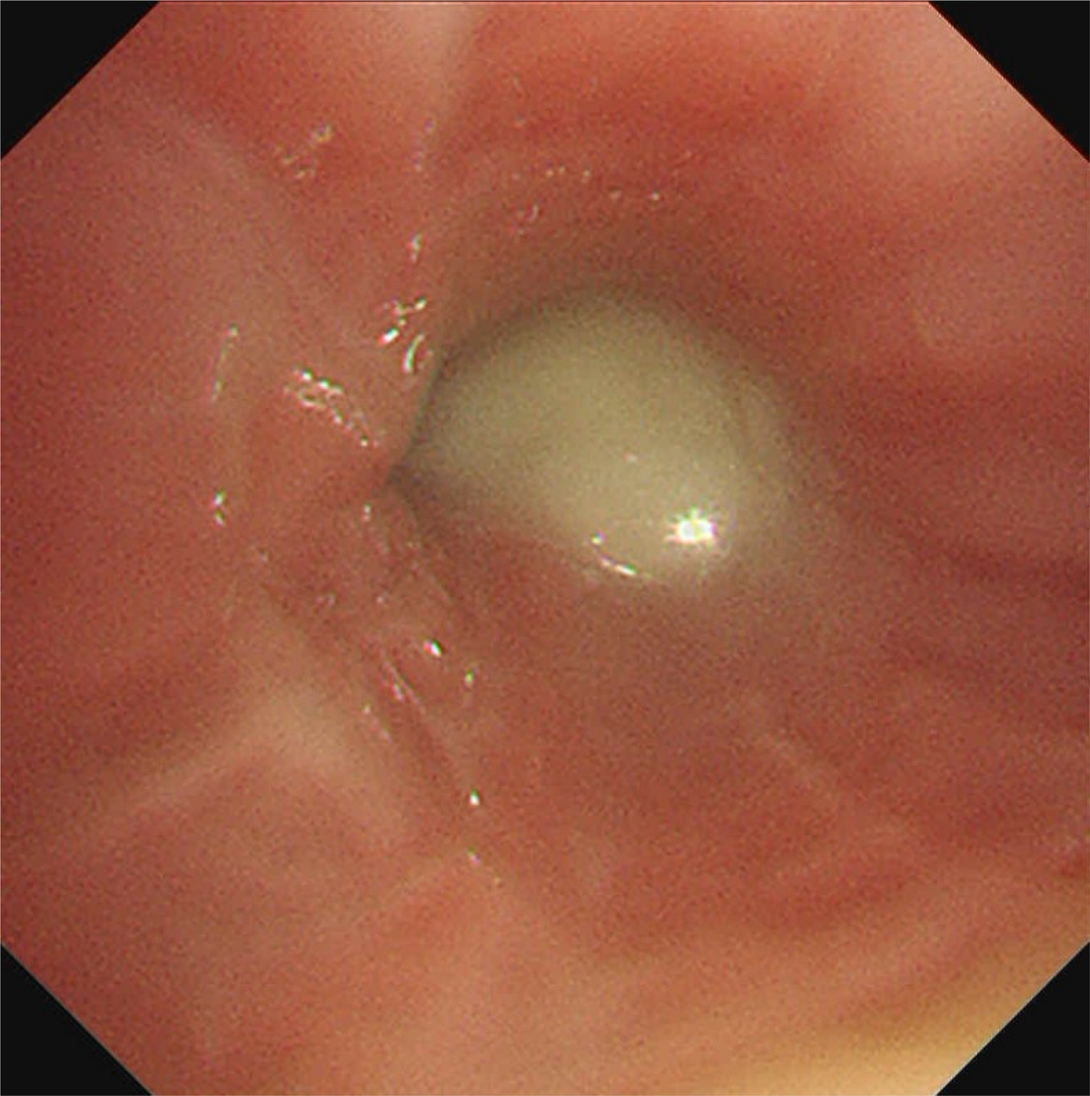
At the first bronchoscopy, the right middle lobe bronchus is difficult to observe from the entrance to the periphery due to purulent secretions.

**FIGURE 4:**
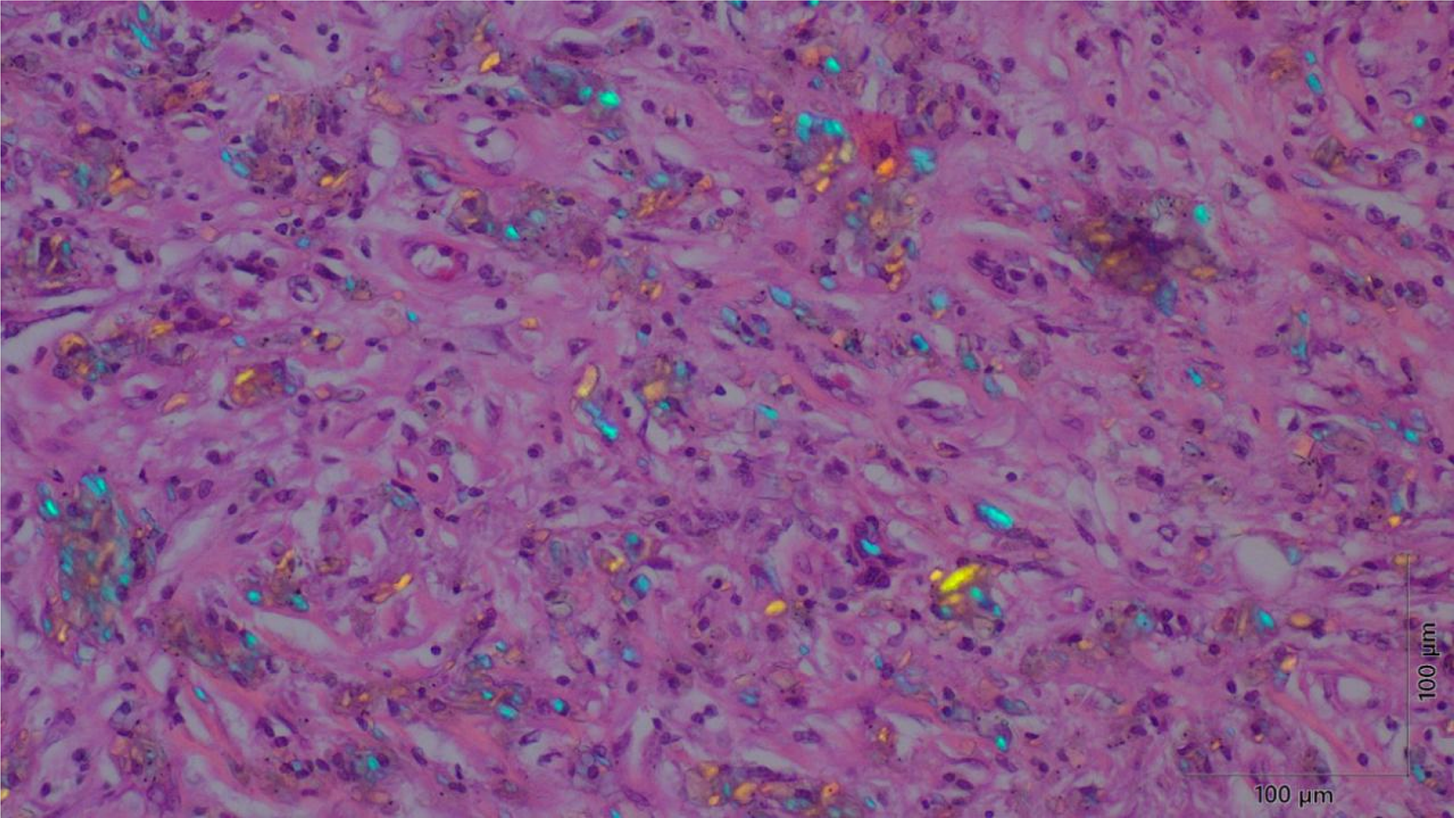
Pathological examination of the excised specimen shows deposition of a fine brown granular material at the site of occlusion (original magnification, 400×; polarized microscope; haematoxylin and eosin staining). These pathological features are similar to those observed in a reaction that normally occurs in the gingiva following leakage of root canal filling.

**FIGURE 5:**
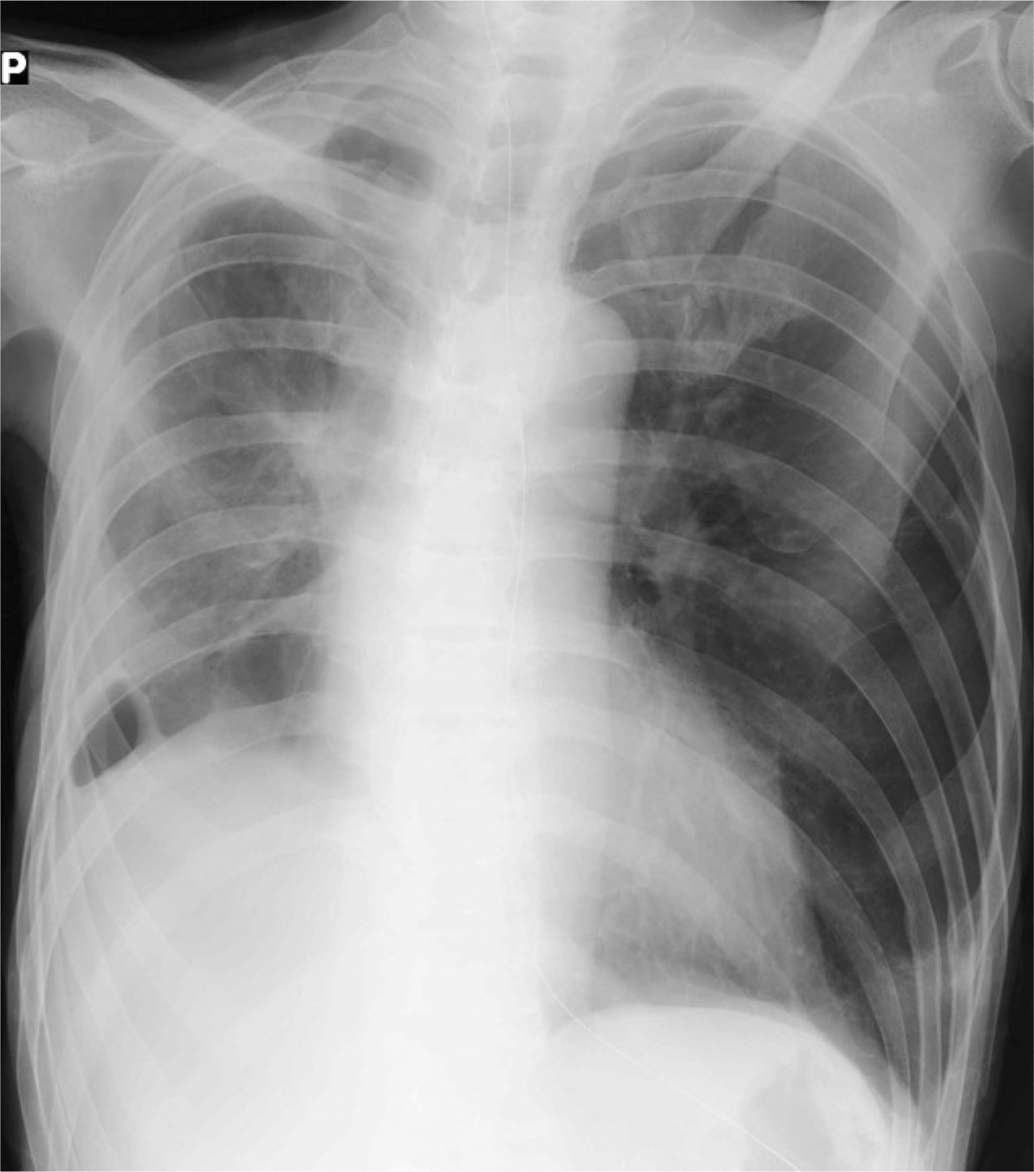
Chest radiograph shows that surgical intervention led to improvement in the infection.

The patient was admitted to the hospital with the diagnosis of a bronchial obstruction due to denture fragment aspiration and consequent obstructive pneumonia. Initially, tazobactam piperacillin (4.5 g/q6h for 14 days) was administered intravenously. Given the anticipated lack of improvement in obstructive pneumonia without removal of the bronchial foreign body, bronchoscopy was performed twice (on the first day of admission and on the seventh day during hospitalization), and attempts were made to remove the foreign body using forceps. However, the foreign body could not be removed because it was not visible (Figure 3) and adhered strongly to the lung tissue. After consulting with the thoracic surgeons, the decision to perform surgical lobectomy was made. Initially, the patient was scheduled to undergo a middle lobectomy; however, the procedure was revised intraoperatively to middle and lower lobectomy due to strong adhesion to the lower lobe and the difficulty of dissection. The resected specimen showed obstructive pneumonia throughout the middle lobe, with fine brown granular material deposits and foreign body granuloma formations at the site of obstruction (Figure 4). In addition, no malignancies were detected. The bronchial foreign body was identified as root canal filling material that is typically used in dental treatment. The pathology in the present case closely resembled that of a root granuloma caused by the leakage of glue‐based root canal filling material. After surgical intervention, the patient became afebrile, and the infection improved (Figure 5). The adverse event was a decreased pulmonary function due to lung resection.

Written informed consent was obtained from the patient for the publication of this case report and all accompanying images.

## DISCUSSION

We encountered a patient with a bronchial foreign body (following denture fragment aspiration) and secondary obstructive pneumonia, who was treated with surgical therapy. The age of predilection for airway foreign bodies is bimodal; the condition occurs in children aged <3 years and adults aged >60 years. Peanuts and other legumes are a common cause of airway foreign bodies in children; dental foreign bodies are more common causes of airway foreign bodies in older adults.^1^, ^2^ Children and older adults may be unaware of this aspiration, and surgical treatment may be considered if removal of the foreign body by bronchoscopy is difficult. Wu et al. reported that the delayed diagnosis of children with bronchial foreign bodies led to significant pulmonary complications, sometimes requiring an open surgery.^4^ In their study, lobectomy was performed in seven patients and pneumonotomy was performed in one patient.^4^ Typically, lung segment removal would be a major undertaking for patients as the last resort. If removal is difficult with a flexible bronchoscope, rigid bronchoscopy or long‐term antibiotic treatment may be considered. However, not all hospitals have specialists skilled in rigid bronchoscopy, and long‐term antibiotic treatment may not be suitable in some cases.

In the present case, the foreign body was difficult to remove using flexible bronchoscopy, and the patient had obstructive pneumonia; this prompted the decision to perform a surgical intervention. Compared to other psychotropic drugs, quetiapine is a weak dopamine antagonist owing to its short binding time to dopamine receptors; however, it reduces substance P levels, resulting in a decreased swallowing function.^5^ Notably, aspiration pneumonia is occasionally encountered as a side effect of quetiapine use in hospitalized patients with delirium. In recent years, many patients have been given easy access to benzodiazepine sleeping pills for insomnia; this poses a serious problem. The side effects of these drugs include muscle relaxation and somnolence, which cause unsteadiness during walking and difficulty in swallowing. In the present case, the patient had a history of manic depression and insomnia and was regularly taking quetiapine and several sleeping pills. These medications caused somnolence and dysphagia, which likely contributed to the aspiration of the denture fragment. Further, he was unable to notice the presence of a bronchial foreign body for a long time because his cough reflex was impaired by these medications.

In the present case, the resected specimen showed obstructive pneumonitis in the entire middle lobe of the lung, deposition of fine brown granular material at the obstruction site, and foreign body granuloma formation. In dentistry, a complication of dental treatment for dental caries is the leakage of the root canal filling material beyond the tooth, which can form a root granuloma. The pathological findings in the present case were akin to the histopathological findings of a foreign body reaction that occurs when root canal filling leaks into the gingiva.^6^ The main component of root canal fillers is zinc oxide (ZnO). Hauman et al. reported that ZnO is cytotoxic in vitro and causes tissue inflammation and granuloma formation.^7^ In the present case, the patient aspirated a dislodged dental foreign body and was unaware of its presence for a long period of time. Leaking components of the foreign body, such as the root canal filling material, may have caused a foreign body reaction in the lungs, which was similar to the reaction that occurs in the gingiva when a ZnO leak occurs.

A unique feature of this case is that the bronchial foreign body (dental root canal fillings) led to granuloma formation at the obstruction site in the late stages, leading to complications that necessitated pulmonary lobectomy. Furthermore, the duration of antibiotic treatment is based on the judgement of the attending physician, and the indications for rigid bronchoscopic removal of the foreign body or eventual pulmonary resection are controversial. We were able to show that relatively early surgery, as in this case, can lead to improvement in the disease.

This case report has some limitations. First, the time from aspiration to obstructive pneumonia is unknown. Second, no attempt was made to remove the foreign body using a rigid bronchoscope due to technical difficulties at our hospital. Therefore, determining when surgery should be performed for such cases is challenging.

In conclusion, when a patient with obstructive pneumonia caused by a bronchial foreign body with unknown aspiration timing is encountered, clinicians should assume that surgical treatment may be necessary. Furthermore, if the foreign body is a root canal filling, the inflammatory reaction can cause it to adhere to the tissue, making its removal using bronchoscopy difficult.

## AUTHOR CONTRIBUTIONS

YY and MU conceived this case report; YY and SY treated the patient; MY and HK interpreted the surgical treatment and pathology results. All authors reviewed the manuscript draft and revised it critically for intellectual content. All authors approved the final version of the manuscript to be published.

## CONFLICT OF INTEREST STATEMENT

None declared.

## ETHICS STATEMENT

The authors declare that appropriate written informed consent was obtained for the publication of this manuscript and accompanying images.

## Data Availability

The data that support the findings of this study are available from the corresponding author upon reasonable request.
